# Metabolic Dysfunction Biomarkers as Predictors of Early Diabetes

**DOI:** 10.3390/biom11111589

**Published:** 2021-10-27

**Authors:** Carla Luís, Pilar Baylina, Raquel Soares, Rúben Fernandes

**Affiliations:** 1 FMUP–Departamento de Biomedicina, Faculdade de Medicina da Universidade do Porto, 4200-319 Porto, Portugal; carlaluis@med.up.pt; 2 i3S-Instituto de Investigação e Inovação em Saúde, Universidade do Porto, 4200-135 Porto, Portugal; rfernandes@eu.ipp.pt; 3 LABMI-PORTIC, Laboratory of Medical & Industrial Biotechnology, Porto Research, Technology and Innovation Center, Porto Polytechnic, 4200-375 Porto, Portugal; pilarbaylina@gmail.com; 4 IPP–Escola Superior de Saúde, Instituto Politécnico do Porto, 4200-072 Porto, Portugal; 5 Biochemistry Unit, Department of Biochemistry, FMUP, Faculty of Medicine, University of Porto, Al Prof Hernani Monteiro, 4200-319 Porto, Portugal

**Keywords:** prediabetes, diabetes, biomarkers, early diagnosis

## Abstract

During the pathophysiological course of type 2 diabetes (T2D), several metabolic imbalances occur. There is increasing evidence that metabolic dysfunction far precedes clinical manifestations. Thus, knowing and understanding metabolic imbalances is crucial to unraveling new strategies and molecules (biomarkers) for the early-stage prediction of the disease’s non-clinical phase. Lifestyle interventions must be made with considerable involvement of clinicians, and it should be considered that not all patients will respond in the same manner. Individuals with a high risk of diabetic progression will present compensatory metabolic mechanisms, translated into metabolic biomarkers that will therefore show potential predictive value to differentiate between progressors/non-progressors in T2D. Specific novel biomarkers are being proposed to entrap prediabetes and target progressors to achieve better outcomes. This study provides a review of the latest relevant biomarkers in prediabetes. A search for articles published between 2011 and 2021 was conducted; duplicates were removed, and inclusion criteria were applied. From the 29 studies considered, a survey of the most cited (relevant) biomarkers was conducted and further discussed in the two main identified fields: metabolomics, and miRNA studies.

## 1. Introduction

According to the International Diabetes Federation, diabetes affects more than 463 million people, with type 2 diabetes (T2D) being the most common, accounting for around 90% of all diabetes worldwide in 2019 [1]. T2D was responsible for more than 4.2 million deaths in 2019, and is also a trigger for other non-communicable diseases, putting considerable pressure on national health systems [1]. T2D is associated with severe comorbidities, such as cardiovascular diseases (ischemic heart disease, myocardial infarction, peripheral arterial disease, heart failure, and stable angina being the most prevalent) [2], kidney diseases (such as glomerulosclerosis and glomerular hypertrophy inflammation/fibrosis, which ultimately lead to diabetic kidney disease) [3], and liver diseases (nonalcoholic fatty liver disease (NAFLD), nonalcoholic steatohepatitis (NASH), liver failure, cirrhosis, and hepatocellular carcinoma) [4], while it also increases the possibility of developing several types of cancer (such as breast cancer, bladder cancer, pancreatic cancer, non-Hodgkin lymphoma, etc.) [5]. Outcomes of such comorbidities can be reduced with early intervention in the development of type 2 diabetes. In recent decades, there has been a massive effort and investment to find biomarkers that can detect T2D early and support the implementation of prophylactic measures.

One of the most important clinical symptoms of diabetes mellitus is hyperglycemia. Thus, monitoring blood glucose levels via a glycated hemoglobin assessment remains the most common screening method. However, when glucose levels are elevated, the disease is already active. Significant investments in research have allowed the identification of biomarkers that can be used to describe the progression from a subclinical to a clinical stage, and some biomarkers have been described as having potential predictive value to differentiate between progressors and non-progressors.

The critical threshold is prediabetes. Prediabetes is an asymptomatic disorder of the normoglycemia–hyperglycemia transitional state, when plasma glucose is above normal range but below clinical diabetes. Prediabetic subjects present either impaired fasting glucose (IFG), impaired glucose tolerance (IGT), or both, as well as an increased risk of developing type 2 diabetes. Such metabolic alterations are already mentioned as being responsible for microvascular problems (such as retinopathy, nephropathy, and neuropathy—persistent complications among the hyperglycemic community) [6]. Whether prediabetes justifies clinical identification and intervention is still continuously debated among international professional organizations, and overall criteria remain without consensus. However, the importance of targeting prediabetes is relevant considering that the risk of developing diabetes can decrease by 40 to 70% with lifestyle alterations in prediabetic patients [7]. The main problem associated with prediabetes is that it may lead to overdiagnosis and, therefore, overtreatment. The pharmacotherapy associated with prediabetes can include antidiabetic drugs such as biguanides (e.g., metformin) or thiazolidinediones (e.g., rosiglitazone), and others, such as GLP-1 analogs or α-glucosidase inhibitors. In addition to pharmacotherapy, bariatric surgery (such as gastric bypass or sleeve gastrectomy) has already been studied in prediabetic patients, with positive results, such as the reversion of IGT to normal values in 98% of individuals [7].

At a prediabetic stage, several metabolic imbalances are already established, occurring before the clinical manifestations. Identifying these imbalances with adequate and precise biomarkers can facilitate early intervention. In America, one in every three individuals have prediabetes, and 11% will develop diabetes [8]. Worldwide, prediabetes is increasing, and the expectation is that, by 2030, the number of people with prediabetes will increase to more than 470 million. Each year, 5–10% will progress to diabetes and develop diabetic comorbidities, such as hypertension [9].

Novel biomarkers can enable the risk stratification of diabetic progression. In this study, a review was performed on new and emerging biomarkers that can act as targets to improve clinical outcomes of the disease’s evolution through early intervention. A study of review articles on the subject was performed to identify the most relevant biomarkers. Among the obtained results, the most cited biomarkers across the studies were further considered and discussed. Our review’s primary objective was to evaluate the research stage and the mechanistic pathway of each biomarker in order to highlight their importance in clinical implementation.

## 2. Materials and Methods

### Search Strategy and Selection Criteria

The present work was developed in 2 steps: the first step aimed to identify the most relevant biomarkers (with review articles); after having identified them, the second step consisted of establishing their respective descriptions.

Initially, a search for English review articles published between 2011 and 2021 (last ten years) was conducted in PubMed. Queries were ‘Biomarkers’ and ‘Prediabetes’/’Impaired fasting glucose’/’Impaired glucose tolerance’. From the collected articles, duplicates and manuscripts with an association between prediabetic condition, impaired fasting glucose, impaired glucose tolerance, and other comorbidities (cardiovascular diseases, cancer, polycystic ovarian syndrome, etc.) or diet-related factors (polyphenols, vegetables, etc.) were removed.

After a thorough assessment of each of the included manuscripts, the biomarkers were identified; those mentioned in a higher number of articles were considered to be the most relevant. Finally, for the most relevant biomarkers, a comprehensive review was carried out regarding description, outcomes, advantages, and disadvantages.

## 3. Results

**Figure 1:**
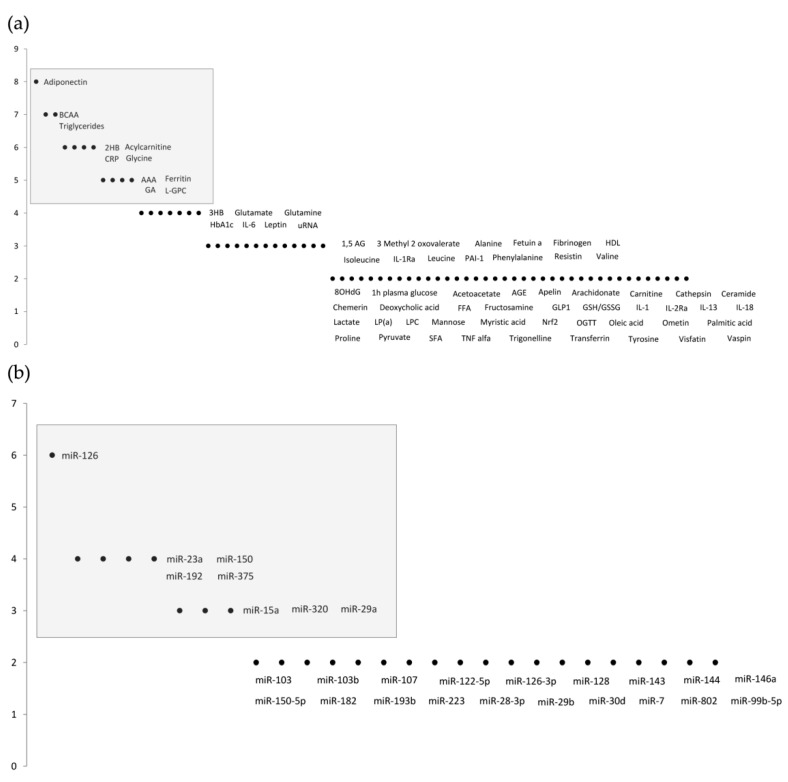
Number of citations regarding biomarkers associated with (**a**) metabolomics studies; (**b**) microRNA studies. The grey areas highlight the most relevant biomarkers. Biomarkers with just one reference were not included in these results.

A total of 145 total cumulative records were retrieved from PubMed, 13 of which were duplicates and, hence, immediately excluded. The title and abstract were examined for the remaining 132 records, following concordance assessment of the inclusion criteria and objectives, resulting in an additional exclusion of 103 records. Thus, 29 studies were identified as being eligible and relevant. All manuscripts were carefully studied, and biomarkers were identified and counted. The analysis of the results identified two approaches to novel prediabetic biomarkers: metabolomics [10,11,12,13,14,15,16,17,18,19,20,21,22,23,24,25,26,27,28,29,30], and microRNA studies [31,32,33,34,35,36,37,38]. The results are shown in Figure 1. Further ahead, Table 1 and Table 2 summarize the most relevant biomarkers’ descriptions, outcomes, advantages, and disadvantages.

### 3.1. Metabolomics Studies

**Figure 2:**
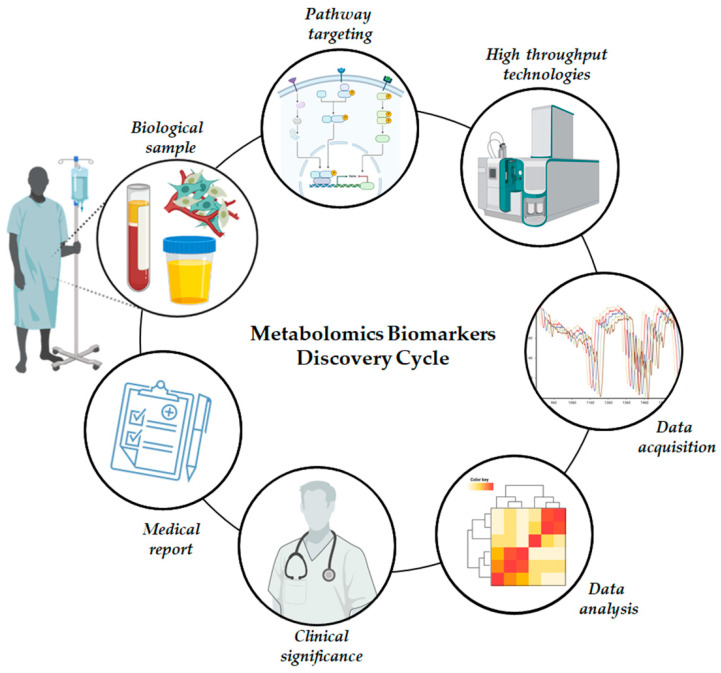
Metabolomics biomarkers’ discovery cycle: from the biological sample, to the identification of the disturbed metabolic signaling pathways, to its clinical significance. Metabolomics results facilitate the integration of the metabolic profile in the pathophysiology of prediabetes.

Metabolomics is a high-throughput technique that enables the identification and quantification of small molecules present in biological samples such as blood, urine, and tissue. Metabolomics is increasingly used to address metabolic dysregulation associated with prediabetes (Figure 2). The method used in metabolomics combines analytical chemistry and data analysis with spectroscopic/spectrometric techniques (such as mass spectrometry or nuclear magnetic resonance) and separation techniques (such as gas chromatography, high-performance liquid chromatography (HPLC), ultra-HPLC, etc.) in a profitable, high-yield manner.

### 3.2. MicroRNA Studies

**Figure 3:**
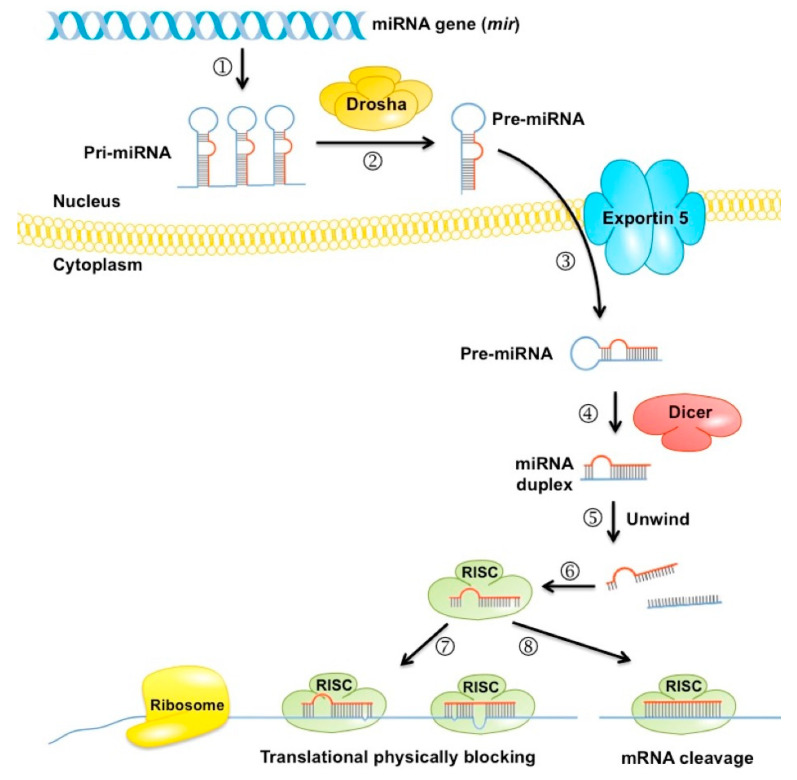
The current model for the biogenesis and post-transcriptional suppression of microRNAs: ① In the nucleus, the miRNA gene is a transcript from RNA polymerase II, which produces a primary miRNA: pri-microRNA (pri-miRNA). ② The pri-microRNA transcripts are first processed into ~70-nucleotide pre-miRNAs by Drosha inside the nucleus. ③ Pre-miRNA is quickly exported by Exportin-5 to the cytosol. ④ In the cytoplasm, the pre-miRNA is processed by Dicer, thus producing a double-ribbon miRNA. ⑤ This product is unwound and then joined with Argonaute to form the complex RISC. ⑥ The RISC complex obtains the pairing between the miRNA and the homolog target mRNA via reverse base complement. ⑦ It subsequently acts on its target through translational repression or mRNA cleavage ⑧, depending, at least in part, on the level of complementarity between the small RNA and its target.

miRNAs are small, non-coding RNAs involved in post-transcriptional gene expression (Figure 3); they can modulate important biological mechanisms such as growth and proliferation, differentiation, and cell death. Research on miRNAs is more recent than metabolomics research, leading to the belief that there is still much to be uncovered. miRNAs are becoming increasingly prominent in many pathologies, including prediabetic studies. Interestingly enough, different miRNA profiles were found in healthy, prediabetic, and diabetic individuals [38].

miRNAs associated with diabetic progression have different types of correlation according to their miRNA-specific function. Moreover, miRNAs can predict diabetic complications such as cardiovascular diseases, chronic renal disease, or retinopathy. They display consistent and reproducible circulating levels, and are stable and resistant to RNase activity—essential characteristics in biomarker assessment. Previous studies concluded that diabetes-related miRNA does not change dramatically in the prediabetic stage [38]. Moreover, due to a wide range of prediabetic-associated miRNAs, choosing a set of representative prediabetic biomarkers is challenging [92,93,94].

## 4. Discussion

Diabetes has already reached global pandemic status, as its incidence ranges all over the world. No longer considered a disease of developed countries, today it is even found that diabetes is becoming more prevalent in developing countries [1]. Investment to find new biomarkers for early detection is of the utmost importance; new and promising molecules have been emerging and achieving improved outcomes.

Glycated hemoglobin (HbA1c) is still the most common screening method to monitor glycaemia status; it is a stable and standardized assay that reflects blood glucose levels over the past 2–3 months. Although commonly used in clinical practice, this method presents notable disadvantages: moderated sensitivity; cutoff inconsistencies; does not consider some variables—such as the production rate and lifespan of red blood cells, body mass index, age, sex, and ethnicity—and it cannot be used in neonatal diabetes; moreover, short-term glycemic changes are not accurate. Regarding prediabetes, there is still no clinical consensus for the HbA1c threshold [95,96].

Another popular screening method is the oral glucose tolerance test (OGTT), which measures glucose clearance of blood taken before and after ingestion of glucose. This is a low-cost, widely accepted (for all types of diabetes), direct method to stratify glucose status. However, it also has some weaknesses in terms of its variability, invasiveness, and time-consuming analysis. Moreover, it is affected by individual variability and other pathologies [66]. Nevertheless, results demonstrate that it is more sensitive for detecting prediabetes than HbA1c [21].

The use of these classic biomarkers to entrap prediabetes was not considered successful enough; hence, new molecules are being considered for use to differentiate diabetic progression to a clinical stage. This study aimed to evaluate the most relevant potential biomarkers, making it possible to determine two types of approaches with different characteristics: metabolomics, and microRNA.

Metabolomic studies aim to better understand the relationships between metabolites and the disease’s pathophysiological mechanisms. Outstanding results have been achieved, although none of the identified biomarkers have yet been implemented for routine diagnostic use in clinical practice. Metabolomics studies are sensitive and rapid, with a high-throughput strategy capable of hundreds of readily achievable analyses, although initial equipment investment may present a challenge [97]. Our results show that the most relevant prediabetic biomarkers in research are 2-hydroxybutyrate, aromatic amino acids, adiponectin, acylcarnitine, branched-chain amino acids, C-reactive protein, ferritin, glycated albumin, glycine, linoleoyl-glycerophosphocholine (LGPC), and triglycerides. Although they do not share a common background, they all exhibit great potential for use as a future clinical diagnostic tool. Some biomarkers are associated with inflammation, adiposity, lipid oxidation, glycation, oxidative stress, and iron metabolism. This is relevant because the metabolic dysregulation observed in diabetic progression can have numerous etiologies. In accordance, we anticipate that the answer may be found in a multiplex set of different biomarkers.

We have also observed new tendencies with microRNA studies. MicroRNAs are present in different types of bodily fluids, such as blood, saliva, and urine. Moreover, they are extraordinarily stable, which renders circulating miRNAs as outstanding candidates for minimally invasive prediabetic biomarkers. Authors seem to agree that optimization should be prioritized, with standardization of the pre-analytical variables (sample collection, isolation, and quantification), alternative biological sources of the sample, and consideration of nonblood biofluids. At the moment, there is no consensus regarding the most promising miRNA multiplex for any pathology [98], including prediabetes. Our study showed that the most relevant miRNAs are miRNA-15a, miRNA-23a, miRNA-29a, miRNA-126, miRNA-150, miRNA-192, miRNA-320, and miRNA-375.

Interestingly, alongside microRNA, one work mentions other potential molecules that can also bring new insights in the implementation of biomarkers: long non-coding RNAs (lncRNAs). These molecules are transcripts with more than 200 nucleotides that are not translated into proteins, although they exhibit pre- and pro-transcription functions. Previous studies suggest that lncRNAs present functionality, but only some lncRNAs’ functional interactions have been previously uncovered [99].

Another potential pathway not contemplated in our study is the gut microbiome—a growing area of interest. The gut microbiome is the totality of microorganisms, bacteria, viruses, protozoa, fungi, and their collective genetic material present in the gastrointestinal tract [100]. Alterations in the microbiome have been associated with different pathologies, including T2D. Previous studies by the University of Gothenburg found alterations in implemented T2D [101] and prediabetes [102]. Alterations in the microbiome are associated with impaired glucose tolerance; however, these results must be interpreted with caution. For example, antidiabetic drugs such as metformin are able to alter the human microbiota; hence, results can be misleading [102].

The authors would like to recognize the limitations of the present study. We found that different manuscripts describe the same class of metabolite in different ways. For instance, some articles consider BCAAs to be one single biomarker, while others describe and consider individual BCAAs. We observed the same in the case of AAAs and FFA. It was impossible to overcome this limitation, but we are aware that this could influence the number of mentioned biomarkers. Moreover, new biomarkers may find themselves at a disadvantage. Additionally, we did not assess publication bias or strength of evidence. As the number of included systematic reviews/meta-analyses was small (4 in 29), we do not consider this an umbrella review.

Global investment is suggested in intervening clinical trials to identify and implement selected molecules as biomarkers for early diagnosis that enable the academic and clinical community to identify patients who will progress to T2D, and immediately address preventive/therapeutic strategies.

## 5. Conclusions

Diabetes is still one of the most challenging health problems worldwide. Programs for prevention and awareness of diabetes have proven to be insufficient to stop this pandemic; hence, clinical intervention could be the answer to avoid diabetic progression by targeting prediabetes. The growing attention to novel glycemic biomarkers is attributable to the limitations demonstrated by both HbA1c and OGTT.

From the present study, our interpretation is that these biomarkers are the ones that, so far, are at a more advanced research stage and, thus, are more promising for clinical implementation. However, many other biomarkers have been the target of research in diabetes (such as ophthalmate or galectin-3) with positive results, demonstrating the continuous effort of the academic community to find, comprehend, and interpret new and reliable molecules for the assessment of the (pre)diabetic pathology.

We believe that a biomarker multiplex is the most effective solution to achieve better sensitivity and specificity in predicting progressors in T2D. Such an achievement would improve patients’ health and decrease the national system’s burden regarding diabetes. Moreover, low-cost, effective interventions in the form of lifestyle changes would be sufficient to diminish drug/surgery-based clinical interventions.

## Figures and Tables

**Table 1:** Prediabetic biomarkers identified in metabolomics studies: description, outcomes, and identification of their advantages and disadvantages.

Biomarker	Description/Outcomes	Advantages/Disadvantages	References
2-Hydroxybutyrate (2HB)	2HB is a metabolite of alpha-ketobutyrate synthesis produced in the threonine and methionine catabolism and glutathione anabolism; it is a predictive marker of hyperglycemia and beta-cell dysfunction; Elevated levels of 2HB are associated with insulin resistance, oxidative stress, lipid oxidation, and diabetic state aggravation. Decreased levels of 2HB were observed 6 months after bariatric surgery as a representative improvement of the pathology.	2HB has proven to be a biomarker independent of sex, age, BMI, and collection site; however, it is still in a premature investigation stage.	[39,40,41,42]
Aromatic Amino Acids (AAAs)	AAAs, tyrosine, and phenylalanine are amino acids with an integrated aromatic ring. Phenylalanine is a precursor of tyrosine, and tyrosine is a precursor of catecholamines. Both tyrosine and phenylalanine are glucogenic and ketogenic amino acids. Increased levels of tyrosine and phenylalanine were observed in obesity-related insulin resistance, and predicted the development of T2D. After diabetic treatment with glipizide and metformin, AAA levels changed in accordance with the patient’s insulin resistance status.	Different expression patterns of amino acids can be predictive of prediabetes in various cohorts. Additionally, significance can be altered after variable adjustment of body mass index (BMI), age, sex, race/ethnicity, and FPG levels.	[18,43,44]
Adiponectin	Adiponectin is a hormone secreted from the adipose tissue with insulin sensitivity, antidiabetic, anti-inflammatory, and anti-atherogenic properties. Adiponectin stimulates a broad spectrum of metabolic actions via ceramidase activation; it is directly correlated with insulin sensitivity, and inversely correlated with T2D development risk. Lower adiponectin levels were observed 10 years prior to T2D diagnosis.	A biomarker independent of ethnic differences, it can be affected by sex-specific mechanisms nevertheless. Certain studies do not corroborate the lower adiponectin levels in prediabetics compared with healthy individuals.	[45,46,47,48]
Acylcarnitine	Acylcarnitines result from the conjugations of acyl-coenzyme A with carnitine conjugation for the transport of fatty acids through the inner mitochondrial membrane for beta-oxidation. They are associated with the NF-κB pathway, and can promote insulin resistance and inflammation. Acylcarnitine has shown to be higher in prediabetes due to the dysregulation of mitochondrial fatty acid oxidation. A panel of acylcarnitines was observed to be associated with T2D development in a 6-year follow-up.	Some acylcarnitines did not show any association with body fat or waist–hip ratio, fat mass, or fat distribution. Overall, they are independent biomarkers of traditional risk factors.	[49,50,51,52]
Branched-Chain Amino Acids (BCAAs)	BCAAs such as leucine, isoleucine, and valine are the most abundant and essential amino acids present in a regular diet. Accumulation of BCAAs activates via mTOR and, consequently, S6 kinase, which leads to serine phosphorylation of the substrate-1 (IRS–1) insulin receptor, causing insulin resistance. High levels of BCAAs are associated with obesity, insulin resistance, impaired glucose tolerance, and T2D. BCAA levels normalize after bariatric surgery.	Phenotypic and genetic factors can influence BCAA levels, which can reveal associations with both sex and BMI. There is still some debate on whether BCAAs are the cause or the effect and, as such, whether they should be considered a biomarker.	[53,54,55]
C-Reactive Protein (CRP)	CRP is an inflammatory biomarker of hepatic origin associated with the acute phase response; it responds to transcription factors released by macrophages and adipocytes. Higher CRP levels were found in patients with prediabetes and insulin resistance, rendering it a sensitive biomarker for early T2D diagnosis. These results may be a consequence of the low state of chronic inflammation grade found before the onset of type 2 diabetes.	Association between CRP and prediabetes is independent of age, sex, ethnicity, alcohol consumption, smoking, hypertension, BMI, and total cholesterol. It is still in an early investigation stage for prediabetes signaling.	[56,57,58,59]
Ferritin	Ferritin is a protein (acute phase reactant) involved in iron storage, which is able to release iron in a controlled manner. Iron contributes to insulin resistance via many pathways, such as β-cell oxidative stress and β-cell apoptosis through ROS formation. Iron metabolism seems to be correlated with T2D status: uncontrolled T2D is associated with iron deficiency. High ferritin levels translate to an increased risk of developing T2D. Dietary restriction and chelation may prevent T2D progression.	The threshold level is still uncertain, and may vary according to age and sex. Ferritin levels are predictive of diabetes progression independently of a comprehensive range of risk factors, such as physical activity, smoking, and family history.	[60,61,62,63]
Glycated Albumin (GA)	Albumin is the most commonly studied soluble protein, and is highly susceptible to post-translational modifications (PTMs). One frequent modification is glycation, resulting in GA. GA plays a vital role in diabetic pathophysiology; it is inversely correlated with obesity and positively correlated with diabetes. The increase observed in diabetes is associated with secondary comorbidities. GA can act as an antigen, elicit the immune response, and form complexes that can accumulate in the arteries and kidneys, leading to nephropathy and atherosclerosis.	Accurate assessment for short-term glycemic control. The enzymatic method is sensitive, fast, and less susceptible to pre-analytical variables. Values of GA are not reliable in individuals with abnormal albumin metabolism.	[22,64,65,66,67]
Glycine	Glycine is a nonessential stable amino acid, able to be synthesized by the body from serine. Glycine is a precursor of protein metabolism, and can act as a neurotransmitter and as a co-ligand for N-methyl-D-aspartate glutamate receptors to control insulin secretion and liver glucose output, functioning on both the pancreas and the brain. Lower glycine levels are associated with an increased risk of prediabetes, type 2 diabetes, and obesity, and are also correlated with insulin resistance and glucose intolerance.	Glycine levels are not dependent exclusively on glycemic status, and may vary in individuals with abnormal amino acid metabolisms or metabolic syndrome.	[10,18,23,68]
Linoleoyl-glycerophosphocholine (LGPC)	Linoleoyl-glycerophosphocholine (LGPC) is a metabolite of the phospholipase A2 hepatic enzyme and lecithin-cholesterol acyltransferase. Known for its anti-inflammatory properties, it acts as a non-competitive enzyme inhibitor of phospholipase A2, usually increasing during the inflammatory state. This metabolite’s plasma concentration is associated with an increased risk of developing insulin resistance, impaired glucose tolerance, and diabetes.	Independent of age, sex, body mass index, familial diabetes, fasting glucose, waist circumference, blood pressure, glycosylated hemoglobin, triglycerides, and high-density lipoprotein cholesterol.	[21,69]
Triglycerides	Triglycerides are the most common lipids present in the body, and are composed of three fatty acids and a glycerol molecule. They are often an indication of conditions such as obesity and metabolic dysfunction. High levels of triglycerides are associated with diabetic progression, beta-cell dysfunction, and impaired insulin secretion. Studies have demonstrated that the product of triglycerides and glucose is able to discriminate prediabetes and diabetes, and triglyceride levels can be improved with physical activity and, therefore, improve glycemic status.	Triglycerides have already been implemented in clinical practice. In prediabetic individuals, high levels of triglycerides are a predictive factor for T2D progression. Studies found variations between different ethnicities.	[70,71,72]

**Table 2:** A brief description and outcomes of miRNAs expression associated with prediabetes, impaired glucose tolerance, and impaired fasting glucose.

miRNAs	Description/Outcomes	References
miRNA-15a	miRNA-15a is associated with several biological processes, such as angiogenesis and insulin production; it is also involved in the activation of TGFβR1, CTGF, and p53 proteins. Lower miRNA-15a levels were found in individuals who developed T2D in a 10-year follow-up. The association between miRNA-15a and diabetic progression was still significant after variable adjustment for age, sex, BMI, and hypertension status.	[73,74]
miRNA-23a	miRNA-23a indirectly targets SMAD4—a critical pathway in the regulation of insulin-dependent glucose transport activity. NEK7 is also a target of miRNA-23a and, in animal models, a low level of NLRP3 induced pyroptosis, mitigating the hepatic and renal complications of T2D. The levels of miRNA-23a are lower in prediabetic and T2D patients compared with healthy individuals. Levels of miRNA-23a can also distinguish prediabetic and T2D patients.	[75,76]
miRNA-29a	miRNA-29a was observed to improve pancreatic beta-cell function in in vitro studies. Likewise, upregulation of miRNA-29a is implicated in diabetic progression by IGT and decreased insulin secretion. Higher expression of miRNA-29a is an independent predictor of T2D, IFG, and IR. Additionally, it is significantly correlated with stress hormone levels.	[77,78]
miRNA-126	One of the most studied miRNAs in prediabetic conditions, it is highly correlated with VEGF, and with the promotion of angiogenesis. Anti-miRNA-126 targets SPRED-1 via Ras/ERK/VEGF and PI3K/Akt/eNOS, inhibiting the proliferation and migration of endothelial progenitor cells and promoting apoptosis. Low levels of miRNA-126 are strongly correlated with the progression of the disease.	[79,80]
miRNA-150	Previous miRNA-150 studies demonstrated its regulatory function in beta-cell formation, hematopoietic stem cell differentiation, and obesity-induced inflammation and insulin resistance by controlling adipose tissue and beta-cell function. In the CORDIOPREV study, prediabetic progressors were evaluated in a 5-year follow-up; miRNA-150 levels were higher in plasma several years before the diagnosis of T2D.	[81,82]
miRNA-192	miRNA-192 is involved in IFG and IGT, triglyceride levels, and the fatty liver index. Moreover, miRNA-192 inhibited the proliferation of pancreatic beta-cell lines and insulin secretion. Levels of miRNA-192 are found to be higher in diabetic subjects. Interestingly, vitamin D supplementation modulates miRNA-192 levels, improving the hyperglycemic status in prediabetic patients.	[83,84,85]
miRNA-320	Expression of miRNA-320 is associated with VEGF, IGF1, and FGF. The VEGFa/miRNA-320 axis modulates proliferation, apoptosis, and angiogenesis of endothelial cells, and has been reported to be an active player in diabetic progression. miRNA-320 is positively correlated with prediabetic incidence, and improves diabetic progression via adipoR1 after duodenal–jejunal bypass.	[86,87,88]
miRNA-375	miRNA-375 is a pancreatic-islet-specific miRNA involved in regulating insulin secretion and maintaining average pancreatic alpha and beta-cell mass. miRNA-375 levels are higher and independently associated in prediabetic and diabetic individuals. Deregulation of miRNA-375 was observed years before the onset of T2D in the CORDIOPREV trial.	[89,90,91]

## Data Availability

No new data were created or analyzed in this study. Data sharing is not applicable to this article.
